# High-Performance Ultraviolet Photodetectors Based on Nanoporous GaN with a Ga_2_O_3_ Single-Crystal Layer

**DOI:** 10.3390/nano14131165

**Published:** 2024-07-08

**Authors:** Junjie Wen, Yuankang Wang, Biao Zhang, Rongrong Chen, Hongyan Zhu, Xinyu Han, Hongdi Xiao

**Affiliations:** School of Integrated Circuits, Shandong University, Jinan 250100, China; junjiewen@mail.sdu.edu.cn (J.W.);

**Keywords:** nanoporous GaN, UV photodetector, DBR, Ga_2_O_3_

## Abstract

The utilization of a nanoporous (NP) GaN fabricated by electrochemical etching has been demonstrated to be effective in the fabrication of a high-performance ultraviolet (UV) photodetector (PD). However, the NP-GaN PD typically exhibits a low light-dark current ratio and slow light response speed. In this study, we present three types of UV PDs based on an unetched GaN, NP-GaN distributed Bragg reflector (DBR), and NP-GaN-DBR with a Ga_2_O_3_ single-crystal film (Ga_2_O_3_/NP-GaN-DBR). The unetched GaN PD does not exhibit a significant photoresponse. Compared to the NP-GaN-DBR PD device, the Ga_2_O_3_/NP-GaN-DBR PD demonstrates a larger light-dark current ratio (6.14 × 10^3^) and higher specific detectivity (8.9 × 10^10^ Jones) under 365 nm at 5 V bias due to its lower dark current (3.0 × 10^−10^ A). This reduction in the dark current can be attributed to the insertion of the insulating Ga_2_O_3_ between the metal and the NP-GaN-DBR, which provides a thicker barrier thickness and higher barrier height. Additionally, the Ga_2_O_3_/NP-GaN-DBR PD device exhibits shorter rise/decay times (0.33/0.23 s) than the NP-GaN-DBR PD, indicating that the growth of a Ga_2_O_3_ layer on the DBR effectively reduces the trap density within the NP-GaN DBR structure. Although the device with a Ga_2_O_3_ layer presents low photoresponsivity (0.1 A/W), it should be feasible to use Ga_2_O_3_ as a dielectric layer based on the above-mentioned reasons.

## 1. Introduction

UV photodetectors (PDs) have found extensive applications in various fields, including optical communication, biological/chemical analysis, high-density photomemory, and environmental monitoring [1,2,3,4]. In practical scenarios, an ideal PD is expected to fulfill the “5S” criteria: high sensitivity, an excellent signal-to-noise ratio, effective spectrum selectivity, a rapid response speed, and strong stability [5]. GaN is recognized as a highly promising material for UV PDs because of its direct wide-bandgap semiconductor properties, which should be attributed to its inherent UV absorption capabilities, high electron saturation drift velocity, excellent radiation hardness, as well as the chemical and thermal stability of GaN materials [6,7]. 

GaN-based UV PDs can be traced back to the 1990s [8]. In order to enhance the photo-responsive properties of the PDs, considerable attention has been paid to GaN nanostructures such as nanowires, nanotowers, and nanopores with a high surface area -to- volume ratio [9,10,11]. Among them, nanoporous (NP) GaN has emerged as a promising candidate due to its simple preparation method (e.g., electrochemical (EC) etching) and low defect density. Compared with GaN nanowire or nanotower PDs, NP-GaN PDs exhibit superior response capability. Generally, the UV -PD based on the NP-GaN presented a high specific detectivity of >10^14^ Jones and high responsivity of >10^4^ A/W [10]. The structures of the above-mentioned PD devices are mostly the metal-semiconductor-metal (MSM) Schottky barrier (SB) type. However, the SB PDs have high leakage currents due to the large thermionic emission currents. To improve this fatal disadvantage, a high SB height at the metal/semiconductor interface should be obtained. Therefore, some insulating layers, such as Al_2_O_3_, SiO_2_, HfO_2_, and Ga_2_O_3_, were inserted between the metal and the semiconductor, thereby forming NP-GaN-based metal–insulator–semiconductor (MIS) PDs [12,13,14,15,16]. Among the insulating layer, the Ga_2_O_3_ layers were obtained by the alternating current bias-assisted photoelectrochemical oxidation of the n-GaN in H_2_O [14] and thermal oxidation of the n-GaN in air [10]. However, the two types of Ga_2_O_3_ layers are amorphous and polycrystalline. 

EC etching can be used to fabricate NP-GaN distributed Bragg reflectors (DBRs), which can be applied in photoelectric devices such as resonant-cavity light-emitting diodes (RCLEDs) [17,18] and vertical-cavity surface- emitting laser diodes (VCSELs) [19,20]. Compared with NP-GaN thin films, in addition to the high surface area to volume ratio, the NP-GaN DBRs had high reflectivity (>99%) in the visible light region and low surface roughness [21]. The high reflectivity means that the PD based on the NP-GaN DBR should have a high UV-to-Visible rejection ratio, while the low surface roughness indicates that the high-quality single-crystal Ga_2_O_3_ film can be grown on the NP-GaN DBR mirror [22]. According to our knowledge, no works about the PDs based on NP-GaN DBRs with and without a Ga_2_O_3_ layer were involved. 

In this paper, we reported two types of novel PD devices based on the NP-GaN DBR with and without a Ga_2_O_3_ single-crystal film. We aimed to reduce the light-dark current ratio, improve the response speed, and enhance the photoresponsivity by inserting a Ga_2_O_3_ layer. Compared with the PD based on the NP-GaN DBR, the NP-GaN DBR/Ga_2_O_3_ PD presented a higher light to dark current ratio, a faster light response speed, and a lower responsivity under 365 nm UV light. Our research lays the foundation for the application of NP-GaN DBRs in PDs.

## 2. Materials and Methods

**Figure 1:**
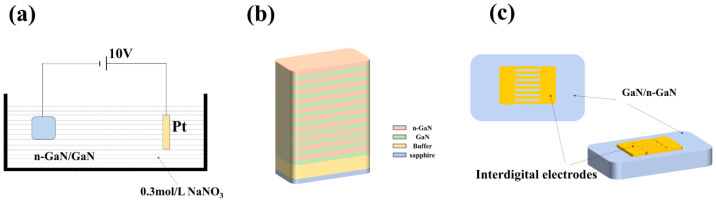
(**a**) EC etching equipment; (**b**) epitaxial growth of λ/4 GaN/n-GaN structures; (**c**) schematic diagram of the GaN-based PD.

To fabricate NP-GaN DBRs, electrochemical etching experiments were conducted in a two-electrode cell at room temperature with unintentional doped (undoped) GaN/n-type GaN periodic structure as the anode, and a platinum wire as the counter electrode in 0.3 M sodium nitrate (NaNO_3_) solution as electrolyte, as shown in Figure 1a. The periodic structure comprising 10 pairs of alternating Si-doped GaNs with a doping concentration (N_D_) of 1 × 10^19^ cm^−3^ and undoped GaN layers was grown on a *c*-plane sapphire substrate using metal-organic chemical vapor deposition (MOCVD) method (see Figure 1b). In the structure, the thicknesses of the undoped and n-GaN layers are 60 and 80 nm, respectively. The etching process was performed at a constant voltage (10 V) for different etching times (20–40 min). 

A MOCVD system was utilized for the fabrication of Ga_2_O_3_ thin films on the DBRs. Throughout the growth process, high-purity oxygen and trimethylgallium were employed as oxide and organometallic sources, respectively. The flow rates for oxygen and trimethylgallium were set at 50 and 1.5 sccm, respectively. The substrate temperature was maintained at 850 °C, and the chamber pressure remained constant at 20 torr for 30 min. Finally, MSM UV PDs were prepared by electron beam evaporation method with interdigital pattern masks using a deposition of 40 nm titanium (Ti) followed by a deposition of 20 nm gold (Au) onto GaN, NP-GaN-DBR, and Ga_2_O_3_/NP-GaN-DBR (see Figure 1c) [23].

The reflectance of DBRs was identified through TU-1901 UV-visible spectrophotometer. The crystalline quality was evaluated by the X-ray diffraction (XRD) θ-2θ and ω scanning. Scanning electronic microscopy (SEM, Nova Nano SEM 450) was used to observe cross-sectional morphologies of the NP-GaN DBR and Ga_2_O_3_/DBR samples, while atomic force microscopy (AFM) was applied to analyze top-view morphology. The time response and *I–V* characteristics of the PDs were obtained under a UV light of 365 nm by a Keithley 2450 source meter.

## 3. Results and Discussion

### 3.1. Potodetector Based on NP-GaN-DBR

#### 3.1.1. Etching Mechanism and Properties of NP-GaN DBR

**Figure 2:**
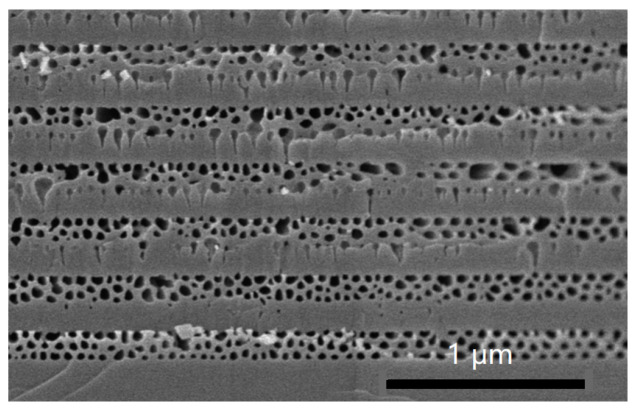
Cross-sectional SEM image of Si-doped GaN (3 × 10^18^ cm^−3^)/Si-doped GaN (1.2 × 10^19^ cm^−3^) periodic structure after the etching.

The recently discovered process of fabricating GaN/NP-GaN DBRs will be briefly discussed here. The EC etching is a selective etching. When a positive anodic bias is used to for GaN samples immersed in an acidic, alkaline, or neutral electrolyte, the GaN is oxidized by the holes created by impact ionization. Subsequently, the oxidized GaN is dissolved in the electrolyte, leading to the formation of nanopores. Figure 2 displays the cross-sectional SEM images of the etched GaN periodic structure under a constant bias voltage. The etching only leads to the formation of very few pores in the lightly doped GaN layers (3 × 10^18^ cm^−3^) and a large number of nanopores in the heavily doped GaN layers (1.2 × 10^19^ cm^−3^). According to the volume average theory [21], the effective refractive index of the porous GaN layer ($n_{eff}$) can be obtained by the Equation (1):

(1) $$n_{eff}={\left[\left(1-\varphi \right)n_{GaN}^{2}+{\varphi n}_{air}^{2}\right]}^{1/2}$$

where φ, $n_{GaN}$, and $n_{air}$ are the porosity, as well as refractive index of GaN and air, respectively. Therefore, the ${\;n}_{eff}$ values of the lightly doped and heavily doped layers are small and large, respectively. To prepare DBRs that can reflect a certain light wavelength (*λ*), the thickness (d) of the GaN and porous GaN layer can be calculated by the Equation (2)

(2) $$d=\frac{\lambda }{4n_{eff}}$$

The reflectivity of the DBR can be obtained by the Equation (3)

(3) $$R={\left[\frac{1-{\left(\frac{n_{H-eff}}{n_{L-eff}}\right)}^{2p}\cdot \;\frac{n_{H-eff}^{2}}{n_{s}}}{1+{\left(\frac{n_{H-eff}}{n_{L-eff}}\right)}^{2p}\cdot \;\frac{n_{H-eff}^{2}}{n_{s}}}\right]}^{2}$$

where $n_{H-eff}$ and $n_{L-eff}$ are the effective refractive index of the porous GaN with high porosity and low porosity, $n_{s}$ is the refractive index of the substrate, and p is the periodic number. For the NP-GaN DBR structure obtained under a constant bias, therefore, the p value increases with the etching time rising, leading to high reflectivity.

**Figure 3:**
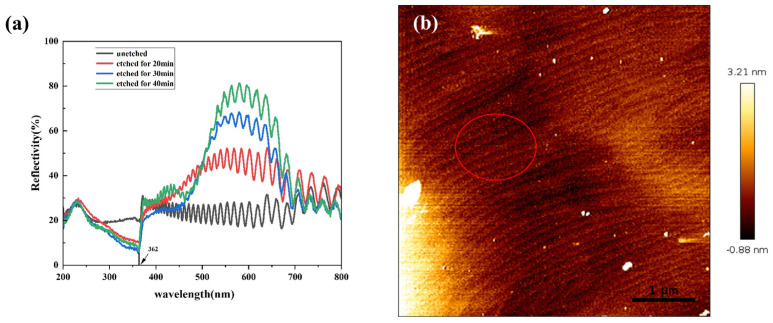
(**a**) Reflectance spectra of the DBRs etched at 10 V in 0.3 M NaNO_3_ solution with different etching times (20–40 min), and (**b**) AFM image of the etched GaN wafer for 40 min.

Figure 3a shows the reflectance spectra of the GaN samples etched at different etching times. All the samples present many interference peaks, which is related to the Fabry-Préot (FT) effect in the top GaN/air and bottom GaN/sapphire interfaces [24]. Compared with the unetched sample with an average reflectivity of 21% in the wavelength range of 200–800 nm, the etched GaN samples present a reflection enhancement almost across the entire visible region (390–690 nm) with the etching time rising, indicating the formation of a DBR structure. For the etched DBR for 40 min, the peak reflectance of >75% is obtained in the reflectance range from 550 nm to 600 nm, and presents a maximum value of 81.1% at 581 nm. More importantly, in the range of 250–380 nm, the reflectance (~20%) of the DBRs is lower than that of the unetched GaN structure. For the DBR (40 min), the reflectivity is less than 10% in the range of 350~365 nm, and reaches a minimum value of 6.2% at 362 nm, indicating that this DBR structure has a stronger absorption capacity for UV light near 362 nm. Figure 3b shows the AFM image of the etched GaN DBR. The whole wafer is very smooth and uniform. The wafer surface shows the presence of a large number of pits (marked by the red circle), which should be the origin of the nanopores in the etched wafer. The root-mean-square (RMS) roughness is 0.98 nm, which is slightly higher than that (<0.5 nm) of the unetched GaN wafer [25], meaning that the DBR can be used as an epitaxial substrate. 

#### 3.1.2. Photoresponse Properties of NP-GaN-DBR PD

**Figure 4:**
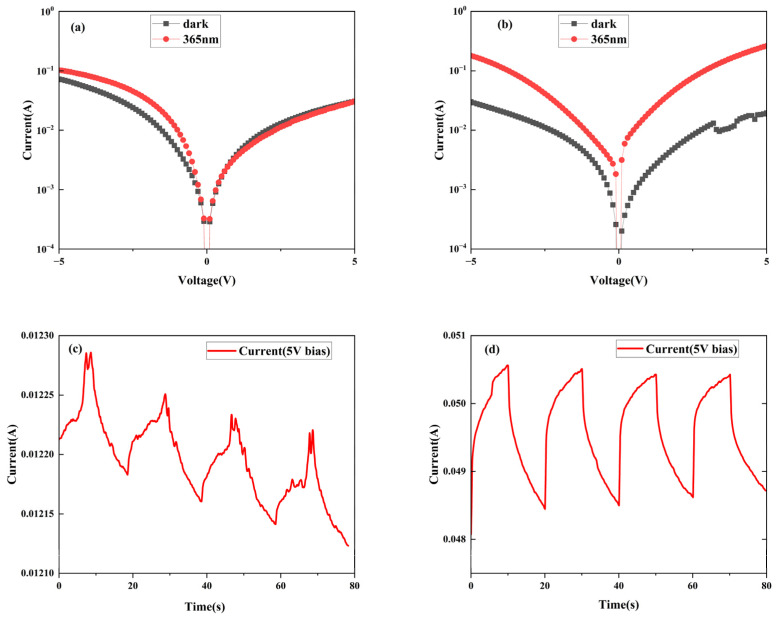
(**a**,**b**) *I–V* characteristics and (**c**,**d**) *I–t* characteristics of the GaN-based PD and NP-GaN-DBR PD under 365 nm.

The NP-GaN DBR structure has enormous potential of for application in light- emitting devices. Due to the ultralow light reflection for 365 nm UV light, the DBR structure should have certain application potential in the UV-light detector. To confirm this hypothesis, interdigital electrodes were deposited on the GaN samples before and after the etching for 40 min to prepare the MSM UV detectors. Figure 4a–d displays the *I–V* and *I–t* curves of the PDs based on the unetched GaN and NP-GaN-DBR. The photocurrent of the unetched GaN wafers is roughly equal to the dark current, making it difficult to fabricate a UV PD (Figure 4a). By comparison, the light-dark current ratio of the NP-GaN-DBR PD at 5 V is increased to ~13 (see Figure 4b), which should be attributed to the DBR PD device with a lower dark current. The EC etching originates from defects such as nano-scale regions of impurity or high carrier concentration. Therefore, while the EC etching leads to a decrease in the carrier concentration, a large number of dangling bonds are formed in the pore wall. Their combined effect leads to a slight decrease in the dark current, resulting in a low light-dark current ratio.

The responsivity (R) of the detectors can be calculated by the Equation (4):

(4) $$R=\frac{I_{light}-I_{dark}}{SP}$$

where I_light_, I_dark_, S, and P are the light -current, dark -current, effective light-sensitive area of the detector, and power density of the UV light, respectively. S is about 0.73 mm^2^, corresponding to the irregular region between the electrodes, P is about 21.5 mW/cm^2^. The calculated responsivity of the NP-GaN-DBR PD at 5 V bias voltage is 12.7 A/W (Figure 4d). Specific detectivity (D*) is another crucial parameter for PDs, which is often used to characterize the minimum detectable signals. Given that the shot noise resulting from the dark current is a significant contributor to the overall noise, the D* value can be calculated using the following Equation (5):

(5) $$D^{*}=\frac{{RS}^{0.5}}{{\left(2eI_{dark}\right)}^{0.5}}$$

Under 365 nm ultraviolet light, the D* value of the NP-GaN-DBR PD is 1.35 × 10^10^ Jones at 5 V bias.

Compared with the unetched GaN-based PD device, the aforementioned improved results can be attributed to two factors. On one hand, the NP-GaN-DBR PD can absorb more UV light at ~365nm, leading to more photogenerated carriers. On the other hand, the photocurrent enhancement is that the defects (e.g., threading dislocation and nano-scale region of impurity or high carrier concentration) in the GaN sample are reduced by EC etching, which greatly reduces the probability of electrons being captured by the defects during the process of excitation from the valence band to the conduction band. The *I–t* curves of the PD devices under UV light with a wavelength of 365 nm at 5 V are shown in Figure 4c,d. Despite exhibiting improved periodicity and a faster response speed compared to the unetched PD device, the NP-GaN-DBR PD device still demonstrates an excessively slow response speed. The combination of this extremely slow response speed and high dark current (10^−2^ A at 5 V) suggests that meeting the application requirements with such a device is challenging.

### 3.2. Photodetector Based on Ga_2_O_3_/NP-GaN-DBR

#### 3.2.1. Characterization of Ga_2_O_3_ Thin Film

**Figure 5:**
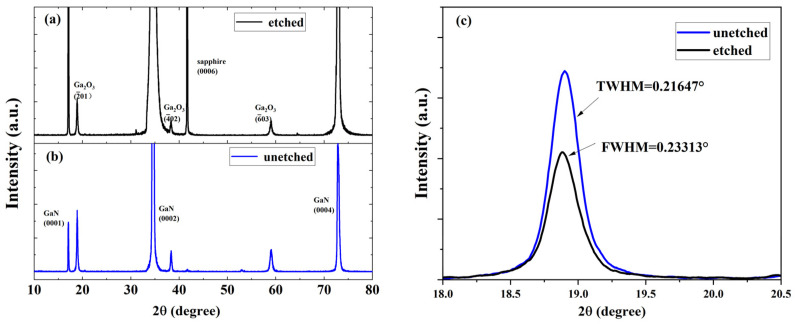
(**a**,**b**) X-ray diffraction (XRD) patterns of Ga_2_O_3_ films grown on the DBR and unetched GaN wafers. (**c**) XRD-ω curves of Ga_2_O_3_($\bar{2}$01) grown on the unetched GaN and DBR substrates.

Figure 5 shows the XRD θ-2θ and ω scanning spectra of the Ga_2_O_3_ films on the GaN wafers before and after the etching. In addition to the diffraction peaks of the substrates located at 17.13°, 34.61°, 72.83°, and 41.72° corresponding to the GaN (0001), GaN (0002), GaN (0004), and sapphire (0006) (PDF#50-0792 and PDF#46-1212), the diffraction peaks centered at 18.91°, 38.36°, and 59.03° correspond to the β-Ga_2_O_3_ ($\bar{2}$01), ($\bar{4}$02), and ($\bar{6}$03) diffraction planes (PDF#41-1103), respectively. This result indicates that the deposited films are grown along a single direction of β-Ga_2_O_3_[$\bar{2}$01]. The rocking curves of the Ga_2_O_3_($\bar{2}$01) planes present good symmetry, indicating that the two films are of a single-crystal structure [26]. The full widths at half maximum (FWHMs) of the rocking curves are 0.233°(Ga_2_O_3_/etched GaN) and 0.216°(Ga_2_O_3_/unetched GaN), representing that the deposited Ga_2_O_3_ single-crystal film on the DBR has lower crystal quality. The lower crystal quality can be attributed to the higher surface roughness of the etched GaN wafer than the unetched GaN wafer [25]. 

**Figure 6:**
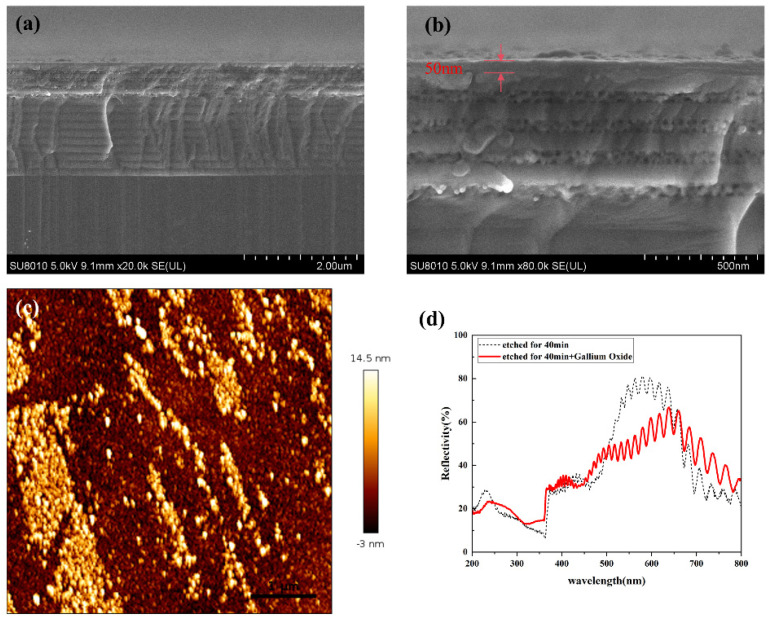
(**a**) Cross-sectional SEM image of the NP-GaN DBR with a Ga_2_O_3_ layer, (**b**) mMagnified SEM image in (**a**), (**c**) AFM image of the NP-GaN DBR with a Ga_2_O_3_ film, and (**d**) reflection spectrum of the NP-GaN DBR with a Ga_2_O_3_ film.

Figure 6a,b displays the cross-sectional SEM images of the Ga_2_O_3_/etched GaN sample. These images reveal the presence of a periodic structure of the GaN/NP-GaN on the etched GaN wafer, providing further evidence for the formation of an NP-GaN DBR structure. Additionally, it can be determined that the grown Ga_2_O_3_ film has a thickness of approximately 50 nm. Figure 6c presents an AFM image (5 μm × 5 μm) depicting the surface morphology of the Ga_2_O_3_ film grown on the GaN wafer after etching. The film surface exhibits numerous islands, and the surface RMS value is 3.98 nm. 

Similar to that of NP-GaN-DBR, the reflection peak of the DBR with a Ga_2_O_3_ film also exhibits interference peaks related to the Fabry-Préot (FT) effect [24]. However, the reflectivity of the latter is significantly reduced over nearly the entire visible region (390–690 nm) (see Figure 6d), probably resulting from the enhancement in the surface scattering due to the increase in the surface roughness (see Figure 6b). In the reflectance range of 250 nm to 380 nm, however, the absorption capacity of the DBR with a Ga_2_O_3_ film decreases, which should be attributable to the reflection of the Ga_2_O_3_ surface with a surface roughness of ~3.98 nm. 

#### 3.2.2. Photoresponse Properties of Ga_2_O_3_/NP-GaN-DBR PD Device

**Figure 7:**
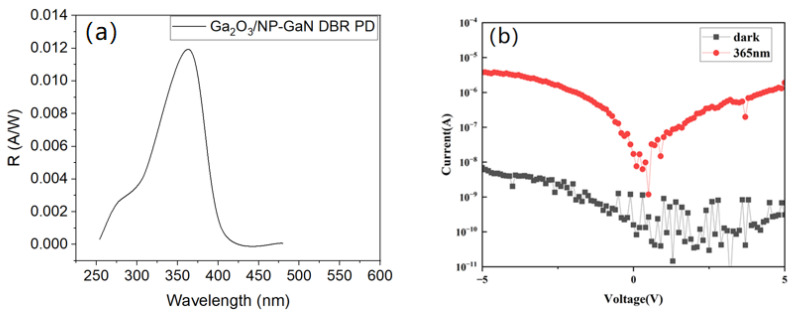
(**a**) Spectral responsivity characteristics and (**b**) *I–V* characteristics of the PD based on Ga_2_O_3_/GaN-DBR.

Figure 7 displays the spectral responsivity and I-V curves of the PD device based on the Ga_2_O_3_/NP-GaN-DBR. Compared with the NP-GaN-DBR PD (Figure S1), the Ga_2_O_3_/GaN-DBR PD presents a similar spectral responsivity curve, which shows the strongest light response around 360 nm (Figure 7a). The photocurrent and dark currents of the PD based on the Ga_2_O_3_/NP-GaN-DBR decrease from 0.26 to 10^−5^ A and from 0.02 to 10^−9^ A, meaning that the specific detectivity and light-dark current ratio increases from 1.35 × 10^10^ to 8.9 × 10^10^ Jones and from 1.30 × 10^1^ to 6.14 × 10^3^ at 5 V (Figure 7b), respectively. The significant decrease in the dark current should be attributable to the implantation of the Ga_2_O_3_ layer between the metal and GaN DBR, leading to higher barrier height [14]. According to the Equation, in addition, the responsivity of the Ga_2_O_3_/NP-GaN-DBR PD at 5 V is reduced to 0.1 A/W, which should be result from the significant decrease in the photo current of the Ga_2_O_3_-based PD. The significant decrease in the photocurrent should be resulted from the fact that most of the photogenerated carriers under reverse-bias are blocked by the Ga_2_O_3_ layer with a thickness of ~50 nm and a bandgap of ~4.9 eV, meaning that only a portion of the holes are transmitted to the metal electrode by tunnels through the defect states within the Ga_2_O_3_ gap. The result is similar with the SiO_2_ layer between the GaN and metal electrode [14]. 

**Figure 8:**
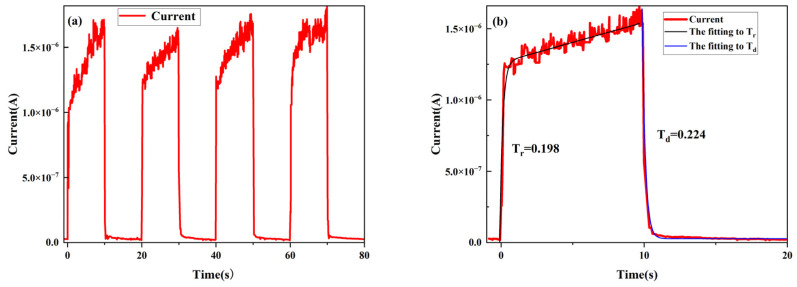
(**a**) Time-dependence light-current characteristics and (**b**) enlarged views of a single light on/off cycle at 5 V bias.

Time-dependent photo response is an important indicator for PDs. Figure 8a shows the time-dependence light-current characteristics of the PD based on the Ga_2_O_3_/DBR under 365 nm light at 5 V bias. The rise (from the dark current to (1 − $\frac{1}{e}$) of the maximum photocurrent during light activation) and decay times (recovering to $\frac{1}{e}$ of the maximum photocurrent during light deactivation) of the Ga_2_O_3_/NP-GaN-DBR PD can be estimated as 0.33 s and 0.23 s, respectively, which are far lower than those (0.85/3.19 s) of the NP-GaN-DBR PD device [27]. This is because the high temperature (850 °C) causes the nanopore walls to become smooth during the growth of the Ga_2_O_3_ [28], leading to the decrease in the tapping density. In addition, we stress the fact that the rise/decay times reported here are one order of magnitude lower than those for the previously reported PDs based on the NP-GaN with a thermal oxidized Ga_2_O_3_ layer [10] that has a rough surface and low crystal quality [29], which should be attributed to the fact that the Ga_2_O_3_ film reported here is a single-crystal film.

## 4. Conclusions

In summary, we successfully fabricated an NP-GaN DBR through electrochemical etching, which exhibited high reflectivity to visible light and absorption to ultraviolet light. The corresponding photodetector demonstrated a high responsivity of 12.7 A/W, a long response time (rise/decay times of: 0.85/3.19 s), and a low light-dark current ratio of 13. Subsequently, we deposited a Ga_2_O_3_ single-crystal film with a thickness of 50 nm on the NP-GaN DBR to create a metal–insulator–semiconductor PD. The dark current of the MIS PD significantly decreased (<10^−9^ A), resulting in an increased light-dark current ratio of 6.14 × 10^3^ and reduced rise and decay times (0.33/0.23 s). Our study provides novel insights into photodetector fabrication utilizing NP-GaN DBRs while further confirming the exceptional photoelectric properties of MIS photodetectors.

## Data Availability

Data underlying the results presented in this paper are not publicly available at this time but may be obtained from the authors upon reasonable request.

## Supplementary Materials

The following supporting information can be downloaded at: https://www.mdpi.com/article/10.3390/nano14131165/s1, Figure S1: Spectral responsivity characteristics of photodetector based on NP-GaN-DBR.

## References

[1] Weisman M.J., Dagefu F.T., Moore T.J., Arslan C.H., Drost R.J. (2020). Analysis of the low-probability-of-detection characteristics of ultraviolet communications. Opt. Express.

[2] Biswal M.R., Arya S., Chung Y.H. (2020). Effect of turbulence and noise on ultraviolet and mid-infrared spectrum in optical wireless communications. Photonic Netw. Commun..

[3] Nasiri N., Jin D., Tricoli A. (2019). Nanoarchitechtonics of Visible-Blind Ultraviolet Photodetector Materials: Critical Features and Nano-Microfabrication. Adv. Opt. Mater..

[4] Ouyang W., Teng F., He J.H., Fang X. (2019). Enhancing the Photoelectric Performance of Photodetectors Based on Metal Oxide Semiconductors by Charge-Carrier Engineering. Adv. Funct. Mater..

[5] Li S., Guo D., Li P., Wang X., Wang Y., Yan Z., Liu Z., Zhi Y., Huang Y., Wu Z. (2019). Ultrasensitive, Superhigh Signal-to-Noise Ratio, Self-Powered Solar-Blind Photodetector Based on n-Ga_2_O_3_/p-CuSCN Core–Shell Microwire Heterojunction. ACS Appl. Mater. Interfaces.

[6] Zhang X., Liu B., Liu Q., Yang W., Xiong C., Li J., Jiang X. (2017). Ultrasensitive and Highly Selective Photodetections of UV-A Rays Based on Individual Bicrystalline GaN Nanowire. ACS Appl. Mater. Interfaces.

[7] Rabiee Golgir H., Li D.W., Keramatnejad K., Zou Q.M., Xiao J., Wang F., Jiang L., Silvain J.-F., Lu Y.F. (2017). Fast Growth of GaN Epilayers via Laser-Assisted Metal–Organic Chemical Vapor Deposition for Ultraviolet Photodetector Applications. ACS Appl. Mater. Interfaces.

[8] Zhang M.-R., Jiang Q.-M., Hou F., Wang Z.-G., Pan G.-B. (2018). Fabrication of high aspect ratio gallium nitride nanostructures by photochemical etching for enhanced photocurrent and photoluminescence property. Scr. Mater..

[9] Guo X.Y., Williamson T.L., Bohn P.W. (2006). Enhanced ultraviolet photoconductivity in porous GaN prepared by metal-assisted electroless etching. Solid State Commun..

[10] Meng R., Ji X., Lou Z., Yang J., Zhang Y., Zhang Z., Bi W., Wang J., Wei T. (2019). High-performance nanoporous-GaN metal-insulator-semiconductor ultraviolet photodetectors with a thermal oxidized β-Ga_2_O_3_ layer. Opt. Lett..

[11] Hu T., Zhao L., Wang Y., Lin H., Xie S., Hu Y., Liu C., Zhu W., Wei Z., Liu J. (2023). High-Sensitivity and Fast-Speed UV Photodetectors Based on Asymmetric Nanoporous-GaN/Graphene Vertical Junction. ACS Nano.

[12] Liu H.Y., Hsu W.C., Chou B.Y., Wang Y.H., Sun W.C., Wei S.Y., Yu S.M., Chiang M.H. (2014). Growing Al_2_O_3_ by Ultrasonic Spray Pyrolysis for Al_2_O_3_/AlGaN/GaN Metal-Insulator-Semiconductor Ultraviolet Photodetectors. IEEE Trans. Electron Devices.

[13] Hwang J.-D., Lin C.J. (2009). High 366/254-nm Rejection Contrast GaN MIS Photodetectors Using Nano Spin-Oxide. IEEE Electron Device Lett..

[14] Lee M.L., Mue T.S., Huang F.W., Yang J.H., Sheu J.K. (2011). High-performance GaN metal-insulator-semiconductor ultraviolet photodetectors using gallium oxide as gate layer. Opt. Express.

[15] Chen C.-H. (2013). GaN-Based Metal–Insulator–Semiconductor Ultraviolet Photodetectors with HfO_2_ Insulators. Jpn. J. Appl. Phys..

[16] Lupan O., Braniste T., Deng M., Ghimpu L., Paulowicz I., Mishra Y.K., Kienle L., Adelung R., Tiginyanu I. (2015). Rapid switching and ultra-responsive nanosensors based on individual shell-core Ga_2_O_3_/GaN:Ox@ SnO_2_ nanobelt with nanocrystalline shell in mixed phases. Sens. Actuators B Chem..

[17] Song Y.K., Zhou H., Diagne M., Ozden I., Vertikov A., Nurmikko A.V., Carter-Coman C., Kern R.S., Kish F.A., Krames M.R. (1999). A vertical cavity light emitting InGaN quantum well heterostructure. Appl. Phys. Lett..

[18] Naranjo F.B., Fernández S., Sánchez-García M.A., Calle F., Calleja E. (2002). Resonant-cavity InGaN multiple-quantum-well green light-emitting diode grown by molecular-beam epitaxy. Appl. Phys. Lett..

[19] Someya T., Werner R., Forchel A., Catalano M., Cingolani R., Arakawa Y. (1999). Room Temperature Lasing at Blue Wavelengths in Gallium Nitride Microcavities. Science.

[20] Lu T.-C., Kao C.-C., Kuo H.-C., Huang G.-S., Wang S.-C. (2008). CW lasing of current injection blue GaN-based vertical cavity surface emitting laser. Appl. Phys. Lett..

[21] Zhang C., Park S.H., Chen D., Lin D.-W., Xiong W., Kuo H.-C., Lin C.-F., Cao H., Han J. (2015). Mesoporous GaN for Photonic Engineering—Highly Reflective GaN Mirrors as an Example. ACS Photonics.

[22] Yang X., Du X., He L., Wang D., Zhao C., Liu J., Ma J., Xiao H. (2020). Fabrication and optoelectronic properties of Ga_2_O_3_/Eu epitaxial films on nanoporous GaN distributed Bragg reflectors. J. Mater. Sci..

[23] Zhu H., Chen R., Han X., Wang Y., Luan C., Ma J., Xiao H. (2024). Fabrication of ZnSnO_3_ single crystal films on sapphire substrates by pulsed laser deposition for solar-blind photodetectors. Appl. Phys. Lett..

[24] Zangooie S., Jansson R., Arwin H. (1999). Ellipsometric characterization of anisotropic porous silicon Fabry-Pérot filters and investigation of temperature effects on capillary condensation efficiency. J. Appl. Phys..

[25] Cao D., Yang X., Shen L., Zhao C., Luan C., Ma J.I.N., Xiao H. (2018). Fabrication and properties of high quality InGaN-based LEDs with highly reflective nanoporous GaN mirrors. Photonics Res..

[26] Chen R., Wang D., Liu J., Feng B., Zhu H., Han X., Luan C., Ma J., Xiao H. (2022). Ta-Doped Ga_2_O_3_ Epitaxial Films on Porous p-GaN Substrates: Structure and Self-Powered Solar-Blind Photodetectors. Cryst. Growth Des..

[27] Liu H., Meng J., Zhang X., Chen Y., Yin Z., Wang D., Wang Y., You J., Gao M., Jin P. (2018). High-performance deep ultraviolet photodetectors based on few-layer hexagonal boron nitride. Nanoscale.

[28] Vajpeyi A.P., Tripathy S., Shannigrahi S.R., Foo B.C., Wang L.S., Chua S.J., Alves E. (2006). Influence of rapid thermal annealing on the luminescence properties of nanoporous GaN films. Electrochem. Solid-State Lett..

[29] Kim S., Kadam M., Kang J.-H., Ryu S.-W. (2016). Optical characteristics of wet-thermally oxidized bulk and nanoporous GaN. Electron. Mater. Lett..

