# Supplementary material for: High-Performance Ultraviolet Photodetectors Based on Nanoporous GaN with a Ga2O3 Single-Crystal Layer
Source: Nanomaterials (Basel). 2024 Jul 8;14(13):1165. doi: 10.3390/nano14131165 (PMC11243192; doi:10.3390/nano14131165)
Supplement: Supplementary file 1 [file nanomaterials-14-01165-s001.zip › nanomaterials-3073703-supplementary.pdf]

Supplementary Materials

High-Performance Ultraviolet Photodetectors Based on Nanoporous GaN with a Ga_2O_3 Single-Crystal Layer

Junjie Wen, Yuankang Wang, Biao Zhang, Rongrong Chen, Hongyan Zhu, Xinyu Han and Hongdi Xiao *

School of Integrated Circuits, Shandong University, Jinan 250100, China; junjiewen@mail.sdu.edu.cn (J.W.)
* Correspondence: hdxiao@sdu.edu.cn

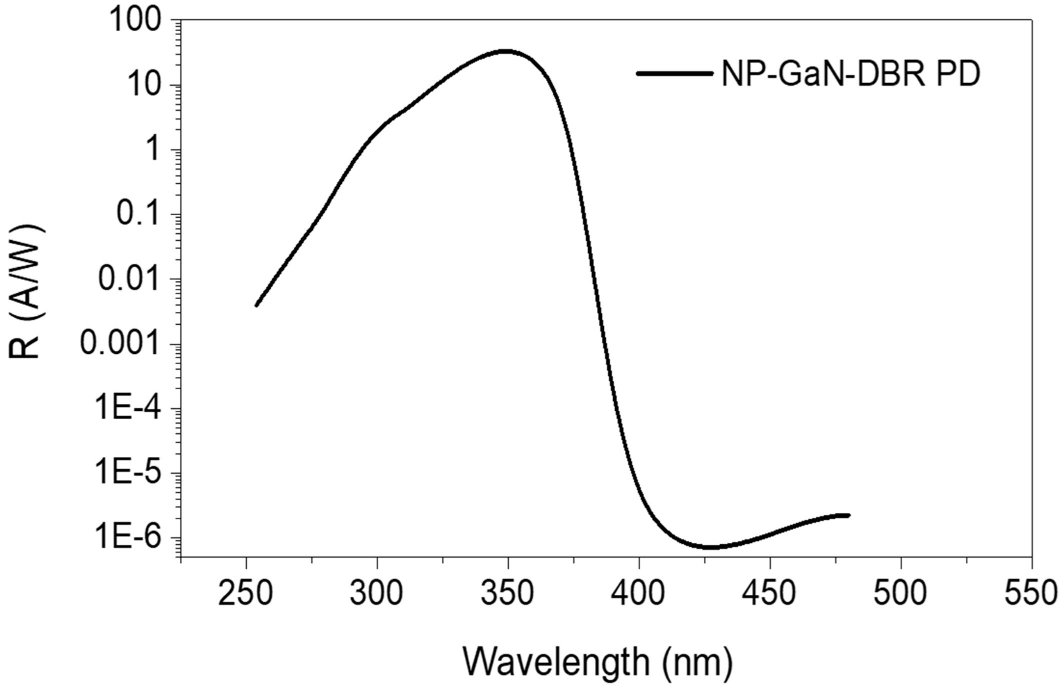

Figure S1. Spectral responsivity characteristics of photodetector based on NP-GaN-DBR.
